# Supplementary material for: Overexpression of the QKI Gene Promotes Differentiation of Goat Myoblasts into Myotubes
Source: Animals (Basel). 2023 Feb 17;13(4):725. doi: 10.3390/ani13040725 (PMC9952742; doi:10.3390/ani13040725)
Supplement: Supplementary file 1 [file animals-13-00725-s001.zip › animals-2170198-supplementary.pdf]

Supplementary Tables

Table S1. Statistical table of sequencing data

Sample	Raw Reads	Raw Bases	Clean Reads	Clean Bases	GC(%)	Q20(%)	Q30(%)
OE1	40,444,350	6.07G	3,941,7024	5.91G	51.32	97.21	92.51
OE3	44,674,980	6.70G	4,358,1784	6.54G	51.84	97.22	92.55
OE2	47,489,000	7.12G	4,601,4404	6.90G	52.33	97.22	92.59
NC1	40,315,362	6.05G	3,921,9804	5.88G	52.22	97.31	92.77
NC2	42,533,996	6.38G	4,147,4496	6.22G	52.21	97.09	92.29
NC3	40,397,998	6.06G	3,877,9502	5.82G	52.24	97.29	92.77

Table S2. Comparisons of transcriptome sequencing data and the reference genome

**Sample**	**Raw Reads**	**Total Map**	**Unique Map**	**Multiple Map**	**Positive Map**	**Negative Map**
OE1	39,417,024	37,915,486 (96.19%)	36,298,110 (92.09%)	1,617,376 (4.1%)	18,120,738 (45.97%)	18,177,372 (46.12%)
OE3	43,581,784	42,031,265 (96.44%)	40,367,617 (92.62%)	1,663,648 (3.82%)	20,150,285 (46.24%)	20,217,332 (46.39%)
OE2	46,014,404	44,364,547 (96.41%)	42,527,659 (92.42%)	1,836,888 (3.99%)	21,226,552 (46.13%)	21,301,107 (46.29%)
NC1	39,219,804	37,885,909 (96.6%)	36,392,892 (92.79%)	1,493,017 (3.81%)	18,163,351 (46.31%)	18,229,541 (46.48%)
NC2	41,474,496	39,942,322 (96.31%)	38,361,063 (92.49%)	1,581,259 (3.81%)	19,144,785 (46.16%)	19,216,278 (46.33%)
NC3	38,779,502	37,372,195 (96.37%)	35,901,018 (92.58%)	1,471,177 (3.79%)	17,914,964 (46.2%)	17,986,054 (46.38%)

Table S3. GO terms related to goat skeletal muscle development

Sample	Description	Up_Gene	Down_Gene
GO:0061061	muscle structure development	3	17
GO:0030016	myofibril	0	17
GO:0043292	contractile fiber	0	17
GO:0007517	muscle organ development	2	14
GO:0060537	muscle tissue development	2	14
GO:0006936	muscle contraction	0	12
GO:0042692	muscle cell differentiation	2	8
GO:0007519	skeletal muscle tissue development	2	6
GO:0055001	muscle cell development	1	7
GO:0014902	myotube differentiation	2	4
GO:0030239	myofibril assembly	0	5
